# A Flexible Method for Genomics‐Based Quantitative Genetics in Wild Study Systems—A Case Study on a House Sparrow Meta‐Population

**DOI:** 10.1111/eva.70242

**Published:** 2026-04-27

**Authors:** Janne C. H. Aspheim, Kenneth Aase, Geir H. Bolstad, Henrik Jensen, Stefanie Muff

**Affiliations:** ^1^ Department of Mathematical Sciences Norwegian University of Science and Technology NTNU Trondheim Norway; ^2^ Gjærevoll Centre Norwegian University of Science and Technology NTNU Trondheim Norway; ^3^ The Norwegian Institute for Nature Research (NINA) Trondheim Norway; ^4^ Department of Biology Norwegian University of Science and Technology NTNU Trondheim Norway

**Keywords:** adaptive potential, Bayesian mixed models, genomic prediction, integrated nested Laplace approximations, micro‐evolutionary change, singular value decomposition, wild study systems

## Abstract

As larger genomic data sets become available for wild study populations, the need for flexible and efficient methods to estimate and predict quantitative genetic parameters, such as the adaptive potential and measures for genetic change, increases. Even though animal and plant breeders, as well as the field of human genomics, have produced a wealth of methods, wild study systems often face challenges due to larger effective population sizes, environmental heterogeneity and higher spatio‐temporal variation. Existing approaches either rely on two‐step procedures, where residuals from a pre‐fitted model are used as the response in a second analysis, or can become computationally inefficient as model complexity and data size increase. We therefore adapt methods from animal breeding to account for the complexity typically present in wild animal populations. The core idea is to approximate breeding values as a linear combination of principal components (PCs), where the PC effects are shrunk with Bayesian ridge regression. The result is a computationally efficient and scalable approach, denoted Bayesian principal component ridge regression (BPCRR). A case‐study for a Norwegian house sparrow meta‐population, as well as simulations, illustrate that the method efficiently estimates the additive genetic variance and accurately predicts breeding values. In order to assess whether BPCRR predicts informative breeding values, we also apply BPCRR to track micro‐evolutionary change across time and space in the house sparrow system. To make the method accessible, we provide coded examples and data.

## 1 Introduction

Understanding how wild populations respond to environmental change requires insight into their adaptive potential. Quantitative genetic methods provide key tools to estimate this potential by focusing on two tasks: (1) estimating the additive genetic variance (*V*_*A*_), the heritable component of phenotypic variation (Falconer and Mackay 1996; Lynch and Walsh 1998), and (2) predicting individual‐specific breeding values (C. R. Henderson 1984; Kruuk 2004). These estimates are crucial in both evolutionary biology and conservation biology (Walsh and Lynch 2018; Kardos et al. 2021), since they allow predictions of adaptive evolution when combined with estimates of selection (Lande 1979; Bonnet et al. 2022). Breeding values have further applications in tracking micro‐evolutionary change and guiding conservation actions (Jensen et al. 2014; Hunter et al. 2022).

For estimation of *V*_*A*_ and prediction of breeding values, relatedness information between any pairs of individuals is traditionally the key information that underlies the statistical models (C. R. Henderson 1984; Kruuk 2004). In wild populations these tasks have traditionally been tackled via the use of long‐term pedigree data using *animal models* (C. R. Henderson 1984; Kruuk 2004; Charmantier et al. 2014), but advances in genotyping have enabled the use of single nucleotide polymorphisms (SNPs) or other genomic data instead. However, when the relatively sparse information on *expected* relatedness from a pedigree is replaced by estimates of *realized* relatedness among individuals from genomic data, the resulting genomic relatedness matrix (GRM) in the *genomic animal model* is very dense (Speed and Balding 2015), which renders statistical model fitting procedures inefficient for large numbers of individuals. Although genomic prediction methods from breeding and human genetics offer many tools (e.g., Meuwissen et al. 2001; Khera et al. 2018; Ødegård et al. 2018; Balding and Speed 2025), wild populations pose distinct challenges: smaller sample size, larger effective population size (*N*_*e*_), environmental heterogeneity, and ongoing evolutionary processes. Despite promising studies (Gienapp et al. 2019; Ashraf et al. 2022; Hunter et al. 2022; Aase et al. 2022), statistical methods have not yet been broadly adapted to these complexities.

An alternative to using the genomic relatedness matrix is marker‐based regression, where breeding values are modeled as the sum of SNP effects (Meuwissen et al. 2001). This is equivalent to the genomic animal model under proper standardization (VanRaden 2008), and is increasingly used in wild systems (e.g., Meuwissen et al. 2016; Ashraf et al. 2022; Hunter et al. 2022). However, because the number of SNPs (*m*) typically exceeds the number of individuals (*N*), regularization methods like BayesA/B/R or Bayesian LASSO are necessary (Park and Casella 2008; Moser et al. 2015). Marker‐based regression scales better computationally with increasing *N* compared to GRM‐based models (Ødegård et al. 2018), but its assumptions often reflect breeding contexts and may not hold in wild populations (Bell 2010). In practice, researchers may simplify models or use two‐step procedures, where residuals from a pre‐fitted model are used as the response in a second model (e.g., Ashraf et al. 2022; Hunter et al. 2022), though their impact on *V*_*A*_ estimates and prediction accuracy remains unclear.

Another, promising solution is to reduce dimensionality using principal component ridge regression (PCRR), which models SNP variation through principal components (PCs) (Ødegård et al. 2018; Selle et al. 2019). Such an approach simplifies prior specification (Gianola 2013) and has previously performed well in a highly inbred cattle population (Ødegård et al. 2018). However, the larger *N*_*e*_ (Palstra and Fraser 2012), potential sub‐structure (Wolak and Reid 2017; Aase et al. 2022), and likely polygenic architecture of traits in wild populations (Goddard et al. 2016) suggest that a relatively high number of PCs might be needed for accurate prediction. Moreover, earlier implementations of PC regression have not included fixed or random effects (Dadousis et al. 2014; Ødegård et al. 2018), limiting their applicability in heterogeneous environments.

Here we propose a flexible and general Bayesian version of the PCRR approach, denoted BPCRR, which flexibly incorporates fixed and random effects, addressing both estimation of $V_{A}$ and genomic prediction for highly polygenic traits in wild systems. We apply this approach to body mass, tarsus length, and wing length in a meta‐population of house sparrows ( *Passer domesticus* ) in Northern Norway—traits known to be fitness‐related and to show spatial genetic and environmental variation (Jensen et al. 2008; Holand et al. 2011; Kvalnes et al. 2017; Araya‐Ajoy et al. 2019). We compare BPCRR to genomic animal models and BayesR in terms of computational efficiency, accuracy, and bias, and demonstrate its utility for tracking micro‐evolutionary change. All Bayesian models were fitted using integrated nested Laplace approximations (INLA) (Rue et al. 2009) via R‐INLA (Martins et al. 2013). BayesR was implemented using the hibayes R package (Yin et al. 2022), which accommodates complex fixed and random effects in a unified framework. In order to reach a wide audience, we provide full code and data for both the simulations and the house sparrow case study.

## 2 Methods

### 2.1 Statistical Modeling Background

#### 2.1.1 The Animal Model and the (G)BLUP

In quantitative genetics, the underlying assumption is that phenotypic characteristics of morphological and life‐history traits have a highly polygenic genetic architecture (Falconer and Mackay 1996; Lynch and Walsh 1998; Goddard et al. 2016), and that inference of the genetic basis of the traits can therefore be made based on similarities between relatives. Variation in complex phenotypes of wild populations has traditionally been additively decomposed using the *animal model* (C. Henderson 1976; Falconer and Mackay 1996; Kruuk 2004), a statistical modeling framework that takes into account the relatedness between the studied individuals and decomposes the phenotype $y_{i}$ of an individual i in its most simple form into a genetic and an environmental component as

(1) $$y_{i}=\mu +g_{i}+\varepsilon_{i},$$

where μ is the population mean, $g_{i}$ is the additive genetic merit (breeding value) for individual i, and $\varepsilon_{i}∼N\left(0,\sigma_{\varepsilon }^{2}\right)$ is the independent environmental effect. The vector of breeding values $g^{⊤}=\left(g_{1},\ldots ,g_{N}\right)$ for N individuals is assumed to follow a multivariate normal distribution $g∼N\left(0,\sigma_{G}^{2}\cdot G\right)$, where G is the relatedness matrix and $\sigma_{G}^{2}$ the additive genetic variance, often denoted as $V_{A}$. The relatedness matrix is either derived from pedigrees, leading to the pedigree‐based animal model and predictions via the BLUP approach (C. R. Henderson 1984), or, more recently, from genomic information, usually from individual single nucleotide polymorphism (SNP) genotype data (VanRaden 2008), which then leads to the *genomic animal model*. In the latter case, the assumption is that marker effects in a large population are uncorrelated (VanRaden et al. 2009), and the G corresponds to the genomic relatedness matrix. The predictions made by this genomic version of the animal model are commonly denoted as genomic BLUPS, or GBLUPs. The animal model (1) is usually extended in various ways in order to capture (permanent) environmental or individual‐specific effects, like sex or age (Wilson et al. 2010).

#### 2.1.2 Marker‐Based Regression

In animal/plant breeding and human genomics, a popular alternative to (both the pedigree‐based and genomic version of) the animal model (1) and its extensions is to formulate a regression model where the phenotype of interest explicitly depends on all the markers, and the breeding value $g_{i}$ is replaced by a sum over the effects of all genome‐wide markers (e.g., Meuwissen et al. 2001; De Los Campos et al. 2010). As for the animal model, the idea is based on the observation that quantitative traits in animals, plants and humans, such as body size, crop yield or disease status, are usually highly polygenic and thus the result of many small contributions from loci across the genome, as well as fixed and environmental factors (Goddard et al. 2016; Walsh and Lynch 2018). For a continuous trait, the *marker‐based* linear model assumes that m SNPs for N individuals are available, and that (in the absence of repeated measurements) the N×1 vector of phenotypes y can be decomposed as a linear combination of contributions from all genomic markers summing up to the breeding value, plus any number of fixed and random effects as

(2) y=μ+Xb+Zu+Wd+ε,

where μ is the vector for the overall mean, X is the matrix containing fixed‐effect variables like sex or age with corresponding regression parameter vector b, Z is an N×m matrix containing the column‐wise mean‐centered marker codes (where the uncentered values typically are 0, 1 or 2 for the *AA*, *AB* and *BB* genotypes, respectively), and $u∼N\left(0,\sigma_{u}^{2}I\right)$ is a random effect for the allele substitution effects of each of the m markers, where I defines the identity matrix of appropriate dimension. Moreover, W is a design matrix of appropriate dimension for all the independent random environmental effects d, and $\varepsilon ∼N\left(0,\sigma_{\varepsilon }^{2}I\right)$ is the vector of normally distributed residuals. The vector of breeding values is then given as g=Zu, and the additive genetic variance as $\sigma_{G}^{2}=\mathrm{var}\left(\mathrm{Zu}\right)$. In the presence of repeated measurements for individuals, the dimensions in the components of model (2) are adjusted accordingly, and an individual‐level *i.i.d*. random effect is added. Equivalence between the genomic animal model and SNP‐based regression given by Equation (2) holds when using the same SNPs, and assuming that effect sizes u all stem from the same distribution (VanRaden 2008). However, note that we only mean‐center, but not variance‐scale the SNPs. While frequency‐based scaling can be useful for emphasizing population structure (Patterson et al. 2006), it may obscure differences in variance that are relevant when the aim is to capture additive genetic variance underlying a trait (Jombart et al. 2009).

In contrast to the genomic animal model, the main problem with model (2) is that we usually have more markers than individuals, that is, N≪m. As a consequence, the SNP design matrix is rank‐deficient, and cannot estimate the effect sizes of each SNP (i.e., the elements in u). Alternative approaches are therefore needed.

#### 2.1.3 BayesR

Bayesian regression models, such as BayesR, address the N≪m problem by placing priors on marker effects that reflect hypotheses about trait architecture (Meuwissen et al. 2001; Habier et al. 2011; Erbe et al. 2012; Moser et al. 2015). Among the “Bayesian alphabet” models, BayesR has consistently shown strong prediction accuracy across a range of traits and architectures (Gianola 2013; Mollandin et al. 2021; Ashraf et al. 2022; Meher et al. 2022). The rationale behind BayesR is that most markers have no or a very small effect, and that the remaining markers affect the trait to different degrees, which is often consistent with what is observed in practice (e.g., Goddard et al. 2016; Yengo et al. 2022). The default BayesR prior on the effect sizes is a Gaussian mixture with zero mean and variances 0, $10^{-4}\cdot \sigma_{G}^{2}$, $10^{-3}\cdot \sigma_{G}^{2}$ and $10^{-2}\cdot \sigma_{G}^{2}$, with Dirichlet priors on the probability vector $\pi =\left(\pi_{1},\pi_{2},\pi_{3},\pi_{4}\right)$ that weights the four components in the Gaussian mixture, and scaled inverse‐chi squared distributions on $\sigma_{G}^{2}$ and $\sigma_{\varepsilon }^{2}$.

A challenge with BayesR is that its original implementation can neither handle repeated measurements, nor random effects other than the genetic value (Moser et al. 2015). The common work‐around to fit models like (2) is to first pre‐adjust the phenotypes for all fixed and random effects using a linear mixed model without a genetic component, and then use the individual‐specific effect (in case of repeated measurements) or the residuals as the new response (Ashraf et al. 2022; Hunter et al. 2022). Even though prediction accuracy is often quite high in those cases, the two‐step approach is arguably not very elegant. In particular, it assumes that there are no genotype‐by‐environment (G×E) interactions, temporal changes in mean breeding values, or interactions with other variables in the model, such as sex or age. In addition, the procedure tends to significantly underestimate $\sigma_{G}^{2}$ (see Appendix A). Here we therefore rely on a recent implementation of BayesR in the R package hibayes (Yin et al. 2022), which can directly handle the full model (2).

### 2.2 The Bayesian Principal Component Ridge Regression (BPCRR) Approach

#### 2.2.1 Dimension Reduction via Singular Value Decomposition (SVD)

A so far under‐explored avenue to tame the number of variables in marker‐based regression in wild systems is via dimension reduction techniques (Ødegård et al. 2018). In the latter case, the SNP matrix Z is decomposed via singular‐value decomposition (SVD), where we obtain an N×N matrix V that contains the eigenvectors. The first k PCs of Z can then be extracted by multiplying the SNP matrix Z by the first k columns of V to obtain a matrix of reduced dimension $Z^{*}=\mathrm{ZV}\left[,1:k\right]$, and reformulate model (2) as

(3) $$y=\mu +\mathrm{Xb}+Z^{⋆}u^{⋆}+\mathrm{Wd}+\varepsilon .$$

Instead of estimating m SNP effects, the problem is reduced to estimating the k PC‐effects in $u^{⋆}={\left(u_{1}^{⋆},\ldots ,u_{k}^{⋆}\right)}^{⊤}$, which is a computationally more manageable problem because k is limited by the number of individuals N. Moreover, the orthogonality of the PCs imposes uncorrelatedness in the PC‐effects, which is at the same time consistent with the assumption of uncorrelated marker effects in model (2) – an assumption that usually is violated for the SNP effects (Macciotta et al. 2010). In addition, the PC‐effects can be assumed to be normally distributed with the same variance (Ødegård et al. 2018; Selle et al. 2019).

The equivalence between the genomic animal model and marker‐based regression (2) implies that the information in the SNPs reflects the relatedness between the individuals, namely because related animals share alleles at a high proportion of genomic markers. As a consequence, we expect that a limited number of principal component (PC)s explain most of the genetic variation, even in a polygenic trait. In fact, experience from animal breeding indicates that very good genomic prediction accuracy can be obtained by using a subset of PCs, usually a number corresponding to about 50%–70% of the number of individuals in a core sample that is representative for the genetic diversity in the population (Ødegård et al. 2018). However, it is not clear to what degree these findings apply to wild systems, with typically higher effective population sizes than the heavily inbred domestic populations, which implies a larger number of independently segregating chromosome segments ($M_{e}$) and thus lower expected linkage disequilibrium (LD) between the SNP markers and quantitative trait loci (QTLs) than in animal breeding (Jensen et al. 2014). The actual choice for the number of PCs k is therefore not trivial and can vary greatly from case to case (Dadousis et al. 2014). Simply speaking, we need to find a balance between over‐ and under‐fitting to accommodate for the bias‐variance trade‐off (James et al. 2013). Here we propose an approach where we combine PC regression with a ridge‐based shrinkage approach. PCs are scaled such that those with smaller variance experience stronger shrinkage, which effectively avoids over‐fitting.

#### 2.2.2 Bayesian Principal Component Ridge Regression (BPCRR)

Simplified versions of model (3) have previously been tackled via frequentist methods, either by using PC regression based on a standard or a partial least squares approach (Solberg et al. 2009; Dadousis et al. 2014), or later via PC ridge regression (PCRR, Ødegård et al. 2018). However, those applications were concerned with data from animal breeding and did not need to consider random (environmental) effects to account for the complexity of wild systems. More specifically, while we usually want to fit the full model (3), previous modeling attempts ignored the Wd component, and sometimes even the fixed effects Xb.

In order to reach full flexibility in our model formulation while enabling efficient computation, we here combine the idea of PC ridge regression (Ødegård et al. 2018) with a Bayesian approach based on INLA (Rue et al. 2009). A previous application from plant breeding indicates that INLA is a suitable and promising candidate for our purpose (Selle et al. 2019). Here we expand the considerations from Selle et al. (2019) in two essential ways: (1) we impose shrinkage on the PC effects, with stronger shrinkage on PCs that explain less variance; and (2) derive a prior that imposes an appropriate level of shrinkage.

##### 2.2.2.1 Scaling the PCs

In ridge regression, all variables are usually standardized to have a variance of one. When working with PCs, however, we have to ensure that components that explain less variance in the SNPs also explain less variance in the response. Since shrinkage is stronger for variables with lower variance (Gianola 2013), we therefore scale the PCs in proportion to their variance explained, which is equivalent to letting the columns of $Z^{⋆}$ have a variance proportional to the corresponding eigenvalue of the SNP covariance matrix (Macciotta et al. 2010). To ensure numerical stability (and without changing the generality of the results), we can then scale (i.e., divide) the variances of all PCs by the standard deviation of the first PC, so that $\mathrm{var}\left({\mathrm{PC}}_{i}\right)\leq 1$ for all PCs. We then set a $u^{⋆}∼N\left(0,\sigma_{u^{⋆}}^{2}I\right)$ prior on the PC‐effects, such that the PCs are identically distributed, with a suitable prior for $\sigma_{u^{⋆}}^{2}$ (which we will discuss below). This, together with the scaled PCs, implicitly assumes that the variance explained by a PC is monotonically related to the variance it explains in the breeding value of the trait of interest, which seems plausible for a highly polygenic trait under the infinitesimal model assumption (see Figure S3 in Appendix B). Note that this way to scale the PCs does not affect the way overall genetic structure of the meta‐population is encoded in the PCs (Figure S4 in Appendix C).

##### 2.2.2.2 Shrinkage Prior for the PC‐Effects

In contrast to the marker‐based Bayesian model, where the choice of the prior on u is critical due to m≫N, PC regression involves fewer variables than data points (k<N) and priors thus become less influential (Gianola 2013). Despite this, it is useful to assign a suitable prior to the variance $\sigma_{u^{⋆}}^{2}$ of the PC effects. To this end, note that the normal prior on $u^{⋆}$ corresponds to a (likelihood‐based) ridge shrinkage factor $\lambda =\frac{\sigma_{\varepsilon }^{2}}{\sigma_{u^{⋆}}^{2}}$ (e.g., Fahrmeir et al. 2004). Let us next look at the BLUP estimator for model (2), sometimes denoted as the SNP‐BLUP (Ødegård et al. 2018). It is known that the respective estimator imposes shrinkage that corresponds to that of a ridge regression with shrinkage factor $\lambda =\frac{\sigma_{\varepsilon }^{2}}{\sigma_{u}^{2}}$, with SNP‐effect variance $\sigma_{u}^{2}=\frac{\sigma_{G}^{2}}{2\sum_{i=1}^{m}p_{i}\left(1-p_{i}\right)}$, where $p_{i}$ is the allele frequency at locus i, meaning that the denominator is the SNP variance summed over all m loci (Gianola 2013; Ødegård et al. 2018). However, this choice of the denominator for $\sigma_{u}^{2}$ is assuming independence between the SNPs, which is problematic due to both LD and the family structure within populations (e.g., Jensen et al. 2014; Uffelmann et al. 2021). The independence assumption is, in contrast, automatically fulfilled for model (3), where the covariates are the (orthogonal) PCs. In order to ensure a corresponding level of ridge‐type shrinkage, we therefore adopt the idea to find the corresponding value for the PC effects variance by scaling the additive genetic variance by the sum of the variances over the k independent PCs that are included in the model

(4) $$\sigma_{u^{⋆}}^{2}=\frac{\sigma_{G}^{2}}{\sum_{i=1}^{k}\mathrm{var}\left({\mathrm{PC}}_{i}\right)}.$$

Note that the idea to leave PC‐variances proportional to the variance they explain in the data implies that PCs with larger variance will be subject to less shrinkage of the corresponding PC‐effect, which is an often overlooked feature of ridge regression (Gianola 2013), and essentially (again) reflects that PCs that explain more variance in the SNP data should also have the opportunity to explain more variance in the response. The Bayesian formulation of model (3) combined with the prior specified in (4) results in an approach that we denote as Bayesian PC ridge regression (BPCRR) with fixed prior assignment. The model, including any additional fixed and random effects, can be fitted in R‐INLA in one step. The ridge‐regression type of shrinkage is thereby only explicitly imposed on the genomic components, while the priors for the remaining parameters can be suitably chosen according to convenience and/or prior knowledge (e.g., Wang et al. 2018, Chapter 5.4.1).

An obvious implication of setting $\sigma_{u^{⋆}}^{2}$ to the value given in (4) is that we need to know $\sigma_{G}^{2}$, ideally without uncertainty. Even though popular existing methods for genomic prediction like BayesR rely on similar prior knowledge (Gianola 2013), the requirement is somewhat circular if the aim is to actually *estimate* $\sigma_{G}^{2}$. Even though it is typically possible to obtain reasonable prior guesses for $\sigma_{G}^{2}$, for example from analyzing a smaller subset or from a previous study, we actually do not need to impose such a “point prior” on $\sigma_{u^{⋆}}^{2}$, but can instead afford to give it a relatively uninformative hyper‐prior thanks to the fact that we are in the k<N regime. To underline this point, we will do sensitivity analyzes by formulating all our models for both the point $\sigma_{u^{⋆}}^{2}$ prior suggested in (4), using a “good prior guess” from earlier analyzes, as well as using the Gamma prior $\sigma_{u^{⋆}}^{2}∼\Gamma \left(1,5\cdot 10^{-5}\right)$, parameterized with shape and rate, which corresponds to the very naive default prior in the R‐INLA framework. The major drawback of using an uninformative prior is that we expect a loss in computational efficiency, as the model has to estimate one extra (hyper‐)parameter.

#### 2.2.3 Optimal Number of PCs for Genomic Prediction

Recall that the quantitative genetic statistical models discussed here have two main purposes: estimation of $\sigma_{G}^{2}$ and the prediction of breeding values. In the former case, the estimated $\sigma_{G}^{2}$ is expected to monotonically increase for an increasing number of PCs (k), where the respective curve asymptotically flattens out once a substantial amount of variance is explained. Note that k≤N, since for k=N (with N being the number of individuals with genotype and phenotype data in the respective analysis), all the variance is explained. On the other hand, if we want to predict breeding values in samples that have not been used to fit the model, careful evaluation of the trade‐off between bias and precision is required, and only an intermediate number of PCs should be used in the modeling procedure in order to avoid under‐ or over‐fitting (Solberg et al. 2009; Dadousis et al. 2014). In principle, the optimal number for k can be found by fitting many models for a dense grid of different numbers of PCs and then choosing the one with highest accuracy. However, such an approach would render the overall procedure inefficient due to the need to repeatedly fit the models for various numbers of k (Solberg et al. 2009). Here we therefore employ theory from animal breeding and human genomics, where a heuristic formula for expected within‐population prediction accuracy has been derived as a function of sample size (N), the number of independent components with estimated effects ($M_{e}$)—usually the number of independent SNP effects—as well as the proportion of variance explained by those components ($h_{M}^{2}$), the SNP‐based heritability for the M SNPs that are included in a particular model

(5) $$E\left(R^{2}\right)\approx \frac{{\mathrm{Nh}}_{M}^{4}}{{\mathrm{Nh}}_{M}^{2}+M_{e}},$$

(Daetwyler et al. 2008; Wray et al. 2013, 2019). Here $R^{2}$ stands for the proportion of phenotypic variance explained in the out‐of‐sample prediction, which is directly related to the expected squared prediction accuracy (i.e., the expected squared correlation between the predicted breeding value and the phenotype, see Section 2.3.2). However, since we operate with k PCs instead of m SNPs, we modify Equation (5) such that $M_{e}$ (which typically is <m) is replaced by k, where $h_{k}^{2}$ denotes the proportion of variance explained in the phenotype by the respective number of PCs

(6) $$E\left(R^{2}\right)\approx \frac{{\mathrm{Nh}}_{k}^{4}}{{\mathrm{Nh}}_{k}^{2}+k}.$$

The rationale is that the $M_{e}$ SNP effects in Equation (5) are assumed independent, which the k PCs fulfill by construction. We thus need to find an approximation for $h_{k}^{2}$, but since we in general (again) want to circumvent the estimation of this quantity for a grid of k due to efficiency reasons, we further assume that $h_{k}^{2}$ is proportional to the variance that the respective PCs explain in the SNPs. This proportionality assumption is plausible for the infinitesimal model, and Figure S3 in Appendix B illustrates its approximate validity for the cases studied here. For a given k, we therefore approximate

(7) $$h_{k}^{2}\approx \left(\frac{\sum_{j=1}^{k}\lambda_{j}}{\sum_{i=1}^{N}\lambda_{i}}\right)h^{2},$$

where $h^{2}$ is the heritability of the trait of interest, which we either assume to be approximately known, or otherwise close to 0.3 (Hansen and Pélabon 2021). Each eigenvalue $\lambda_{j}$ of the SVD corresponds to the SNP‐variance explained by the respective PC, thus $h_{k}^{2}$ corresponds to $h^{2}$ multiplied by the proportion of variance explained by the first k PCs. For a given set of SNPs (and their corresponding SVD), the optimal k thus only depends on the sample size and the heritability of the trait, given that the imposed assumptions are approximately correct.

Importantly, we must recall that the BPCRR approach imposes shrinkage on the PC effects, with stronger shrinkage on PCs that explain less variance. Scaling the PCs as suggested here thus prevents over‐fitting with increasing k, and we do therefore not expect a significant decrease in prediction accuracy even when adding a larger number of PCs than the “best” k found via Equation (6). The respective k should thus be seen as a lower limit, and not necessarily as the final choice. However, given that computational efficiency is a limiting factor, choosing k as small as possible is desired, as unnecessarily large k does not lead to improved accuracy.

### 2.3 The House Sparrow Case Study

#### 2.3.1 The Data Set

All methods were tested and validated with empirical data from a long‐term study on house sparrows in an archipelago at the coast of northern Norway (Jensen et al. 2008; Araya‐Ajoy et al. 2019). We used data on adult house sparrows present on eight islands in the study system during the breeding seasons (May–August) 1998–2012 (Niskanen et al. 2020). Birds were marked with a numbered metal ring and a unique combination of plastic color rings for later individual identification. Some individuals were ringed in the nest and only observed as adults, whereas others were captured with mist nets as adults and then measured for morphological traits such as tarsus length (to nearest 0.1 mm), wing length (to nearest mm) and body mass (to nearest 0.1 g) (Kvalnes et al. 2017). Because house sparrows are sedentary and only 22% perform short‐distance natal dispersal, some adult individuals were captured and measured multiple times during their lives, either on the island they were born or on a neighboring island (Saatoglu et al. 2021). From all ringed individuals a small blood sample (25 μL) was taken to obtain DNA (Lundregan et al. 2018; Niskanen et al. 2020), which was the basis for individual genotyping of SNP markers distributed across most chromosomes in the house sparrow genome by using a custom Axiom 200 K SNP array (Lundregan et al. 2018). After quality control, genotype data on 182,848 polymorphic SNPs were available for 3032 adult individuals (Lundregan et al. 2018; Niskanen et al. 2020). Less than 0.6% of all SNP‐genotypes were missing, and those were mode‐imputed.

#### 2.3.2 Statistical Modeling

We compared the BPCRR method to the genomic animal model and BayesR (see Sections 2.1 and 2.2), in order to understand whether or how the differences in the assumptions affect the results, and to benchmark their efficiency. To this end, we estimated $\sigma_{G}^{2}$ and assessed the accuracy of genomic prediction in the house sparrows for the three continuous traits body mass, tarsus length and wing length. Among the genotyped individuals, we had phenotypic measurements for N=1918, 1915 and 1912 individuals on each of these traits, respectively. About half the individuals had only one measurement for each trait, roughly 25% had two, and the remaining individuals had three or more (maximum 13) measurements. The total number of measurements were 4249 for body mass, 4368 for wing length and 4373 for tarsus length. All our models, based on Equation (3), included as fixed effects X the individual's sex (1 for male, 0 for female), the genomic inbreeding coefficient $F_{\mathrm{GRM}}$ (Niskanen et al. 2020), age at the time of measurement (in years), and the month when the measurement was performed (May to August, quantitatively encoded as 5, 6, 7 and 8 and modeled as a continuous predictor). Since the house sparrow study system comprises a genetically heterogeneous meta‐population, we also included variables reflecting proportional genetic origin from three major genetic groups (inner, outer and other islands) to avoid biased estimates of $V_{A}$ as well as predicted breeding values, as described elsewhere (for more details, see Muff et al. 2019). The permanent individual effect, the island where the individual was measured, and the year of the measurement were modeled as additional independent Gaussian random effects, as encoded in the matrix W of Equation (3).

##### 2.3.2.1 Estimation of Additive Genetic Variance

To investigate the quality of the estimation for $\sigma_{G}^{2}$ using the BPCRR method to approximate the genomic component $Z^{⋆}u^{⋆}$ in Equation (3), we used both a fixed prior for the PC effects variance $\sigma_{u^{⋆}}^{2}$ as given in (4) with a trait‐specific prior estimate of $\sigma_{G}^{2}$ (denoted from now on as *BPCRR fixed*), as well as for the vague default $\sigma_{u^{⋆}}^{2}∼\Gamma \left(1,5\cdot 10^{-5}\right)$ Gamma prior in INLA (denoted as *BPCRR default*), as described in Section 2.2.2. Models were fitted with different numbers of PCs starting with k=100 and increasing in steps of 100 to obtain a grid of *k*‐values. The expectation is that the estimated $\sigma_{G}^{2}$ increases with k, but that the curve flattens out when most of the genetic variance in a given trait is explained by the PCs. For comparison, we also provide independent assessments of $\sigma_{G}^{2}$ from established methods, namely the genomic animal model and BayesR. Both BPCRR and the genomic animal model were fitted in a Bayesian framework using R‐INLA to derive the marginal posterior distributions, where we assigned independent $N\left(0,10^{4}\right)$ priors to all fixed effects and penalized complexity priors (Simpson et al. 2017) $\mathrm{PC}\left(1,0.05\right)$ to the variances of the remaining random effects (i.e., for all except $\sigma_{u^{⋆}}^{2}$). For BayesR, we used the R package hibayes (Yin et al. 2022) with data‐inferred hyper‐parameters for the inverse Chi‐square distribution that is assumed for the marker effects, as described in Yin et al. (2022), although the results are expected to be insensitive towards the actual choice of the prior (Ashraf et al. 2022).

##### 2.3.2.2 Genomic Prediction

We evaluated the prediction accuracy of the posterior means from BPCRR for the number of PCs found by optimizing Formula (6), as well as for k=50, 100, 200, 500, 1000, 1500 and N (the maximum number of PCs for each trait), both for fixed $\sigma_{u^{⋆}}^{2}$ and R‐INLA default priors, via a 10‐fold cross‐validation (CV). Note that folds were defined by selecting individuals and not single measurements, to avoid having individuals appear in several folds. Here, prediction accuracy was reported as the correlation between the predicted breeding values $\hat{g}$ and the mean phenotype $\bar{y}$ over all the repeated measurements per individual $\mathrm{cor}\left(\hat{g},\bar{y}\right)$. We expect this to be in good (although not exact) agreement with the predicted accuracy $\sqrt{E\left(R^{2}\right)}$ from Formula (5), which predicts $\mathrm{cor}\left(\hat{g},y\right)$, while we replace y by $\bar{y}$ due to the presence of repeats. Moreover, note that our way to report prediction accuracy deviates from the scaling $\mathrm{cor}\left(\hat{g},y\right)/h$ that often is used in other work (e.g., Ashraf et al. 2022), but for the methods comparison done here, we decided to work on the scale of the theoretically predicted accuracy of Formula (5). The results were benchmarked against the GBLUPs from the genomic animal model and predictions from BayesR, both in terms of prediction accuracy and bias. We assessed the predictive ability of the different methods by reporting the bias of the genomic prediction results. To this end, we regressed the mean observed phenotype per individual against the predicted breeding values and reported the actual slopes $\beta_{1}$. If the predicted breeding values are unbiased, the expected value for $\beta_{1}$ corresponds to 1.0 (Meuwissen et al. 2001). Finally, all methods were compared with respect to computation time.

#### 2.3.3 Tracking Micro‐Evolution Across Time and Space

In order to not only assess whether BPCRR gives high prediction accuracy compared to existing methods, but also predicts breeding values that yield informative and relevant patterns that are consistent with existing knowledge of a real system, we employed the house sparrow case study to investigate micro‐evolutionary change over time, as well as quantitative genetic population differentiation across space for the three traits. To this end, we grouped the posterior means of the breeding values for each individual according to year of birth (i.e., cohorts from 1997 to 2012) or according to their natal island, respectively (Niskanen et al. 2020; Saatoglu et al. 2021). In order to quantify micro‐evolutionary change over time, we regressed the estimated breeding values against birth year. Furthermore, to ensure that uncertainty in the regression was properly accounted for, we also sampled breeding values from the posterior distribution for each individual and regressed these against birth year, which we repeated 1000 times in order to obtain a posterior distribution for the regression slopes (Morrissey et al. 2014). Here all breeding values were estimated from BPCRR including 1900 PCs, because the goal was within‐sample estimation and not out‐of‐sample prediction.

### 2.4 Simulation

Based on the set of existing SNPs from the house sparrow meta‐population, we carried out a simulation study where we generated phenotypes for all N=3032 individuals in the SNP dataset according to model (2), but without any fixed or random effects $\left(\mathrm{Xb}=\mathrm{Wd}=0\right)$. Assuming a genetic architecture which reflects that marker‐effects in complex traits often have a somewhat skewed distribution with a few high‐effect markers and more markers with low to no effect (e.g., Yengo et al. 2022; Gauzere et al. 2023), we sampled the SNP effects ($u_{j}$) for a hypothetical polygenic trait from a mixture of four zero‐mean normal distributions as

$$u_{j}∼\pi_{1}\cdot 0+\pi_{2}N\left(0,\sigma_{G}^{2}10^{-4}\right)+\pi_{3}N\left(0,\sigma_{G}^{2}10^{-3}\right)+\pi_{4}N\left(0,\sigma_{G}^{2}10^{-2}\right),$$

with $\pi^{⊤}=\left(\pi_{1},\pi_{2},\pi_{3},\pi_{4}\right)=\left(0.95,0.04,0.008,0.002\right)$, indicating that each effect $u_{j}$ stems from either of the four distributions with the probabilities given in the π vector. Using the respective set of markers $u_{1},\ldots ,u_{m}$, we then generated a set of breeding values as a linear combination $g_{i}=\sum_{j}x_{\mathrm{ij}}\cdot u_{j}$, where $x_{\mathrm{ij}}$ denotes SNP j for individual i. The set of $g_{i}$ were then scaled such that $\sigma_{G}^{2}=0.33$, and phenotypes were generated as $y_{i}=g_{i}+\varepsilon_{i}$ with $\varepsilon_{i}∼N\left(0,\sigma_{\varepsilon }^{2}\right)$ using $\sigma_{\varepsilon }^{2}=0.67$, in order to attain a heritability of $h^{2}=0.33$. For both the estimation of $\sigma_{G}^{2}$ and genomic prediction, we carried out the same comparisons and benchmarking as for the analysis of the empirical house sparrow example. Note that, since the simulated data actually are generated according to the underlying modeling assumptions of BayesR (Erbe et al. 2012; Gianola 2013), that approach has an advantage compared to the other models. An additional small simulation with various genetic architectures, but using a mixture of only two components in the distribution of SNP‐effects, is given in Appendix D.

### 2.5 Computational Details and Software

All computational details and information about used software is given in Appendix E.

## 3 Results

### 3.1 Estimation of Additive Genetic Variance

**FIGURE 1:**
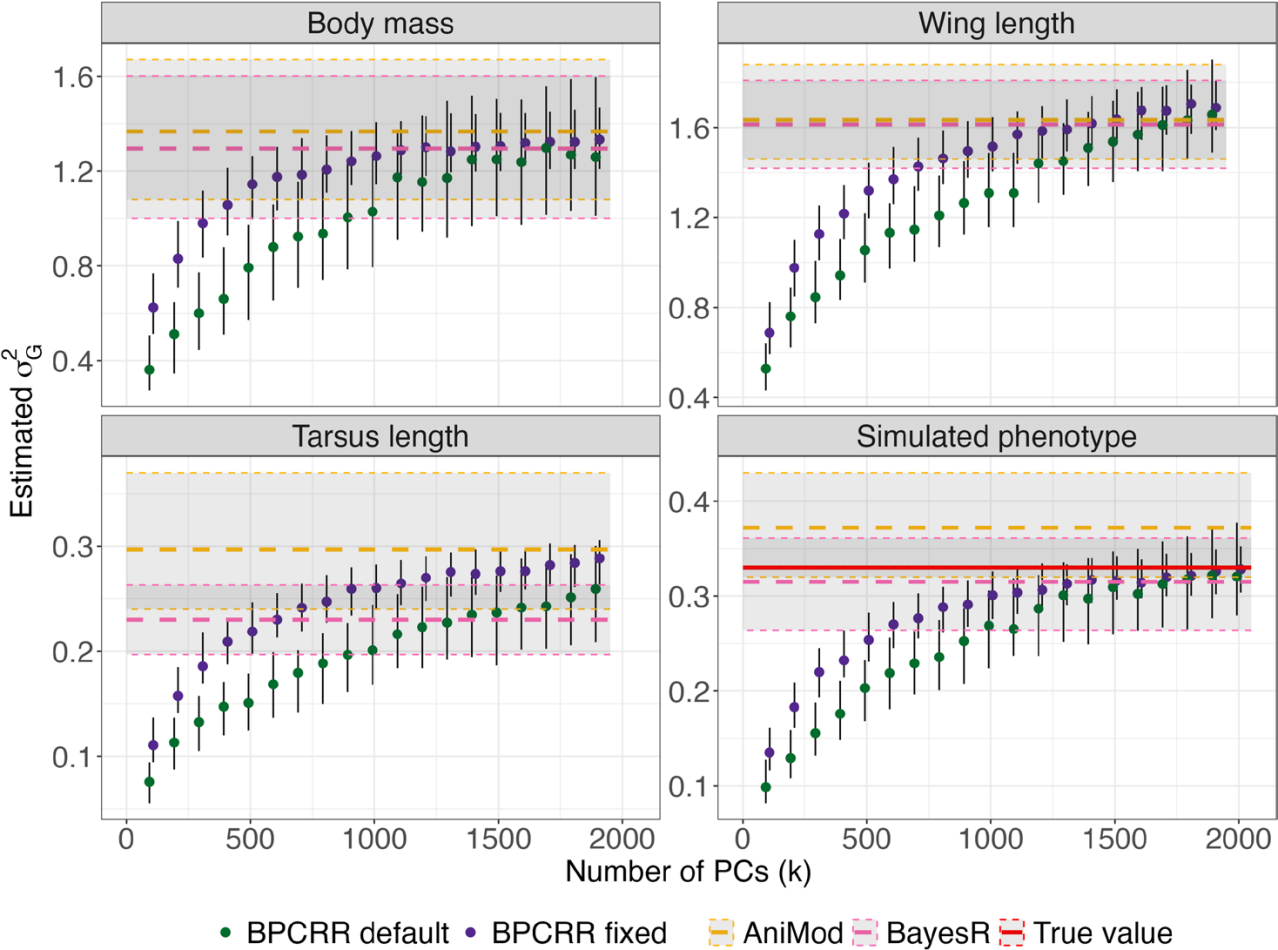
Estimates of $\sigma_{G}^{2}$ using BPCRR dependent on the numbers of PCs (with fixed and INLA default priors in purple and green, respectively), BayesR (in pink) and the genomic animal model (AniMod, in orange) for the three traits from the house sparrow example, as well as for the simulated example. Vertical error bars and horisontal shaded areas correspond to 95% credible intervals. The solid red line corresponds to the true value for the simulated phenotype.

As expected, the estimated $\sigma_{G}^{2}$ for the three traits body mass, tarsus length and wing length from the house sparrow case study, as well as for the simulated case, increased with an increasing number of PCs included in BPCRR (Figure 1). In all cases, the increase in estimated $\sigma_{G}^{2}$ asymptotically flattened out and converged towards the final value, but this happened in different ranges for the different traits and depended on the actual prior used in BPCRR (Figure 1). As expected, when using R‐INLA's default prior for BPCRR, more PCs were needed for convergence and the 95% credible intervals (CIs) were a bit wider than for the informative (fixed) prior derived in Equation (4). For fixed prior variance on $\sigma_{u^{⋆}}^{2}$, 1000 PCs typically resulted in good approximations for $\sigma_{G}^{2}$, whereas more PCs were needed when uninformative priors were used.

The results of BPCRR, BayesR and the genomic animal model are generally in good agreement. For tarsus length, however, BayesR predicted a somewhat smaller and the genomic animal model a larger $\sigma_{G}^{2}$ compared to BPCRR, even when all PCs were included. Similarly, the genomic animal model overestimated $\sigma_{G}^{2}$ in the simulation study. In addition, $\sigma_{G}^{2}$ for tarsus length was not converging for any k below the maximum number of PCs for BPCRR. These observations may indicate that the assumption for the genomic animal model, namely that the marker effects stem from one normal distribution, are violated. In fact, previous studies found that leg bone traits in other species have a more oligogenic architecture than other traits (e.g., Ashraf et al. 2022), and the simulated trait was constructed similarly.

### 3.2 Genomic Prediction

#### 3.2.1 Selecting the Number of PCs in BPCRR

**FIGURE 2:**
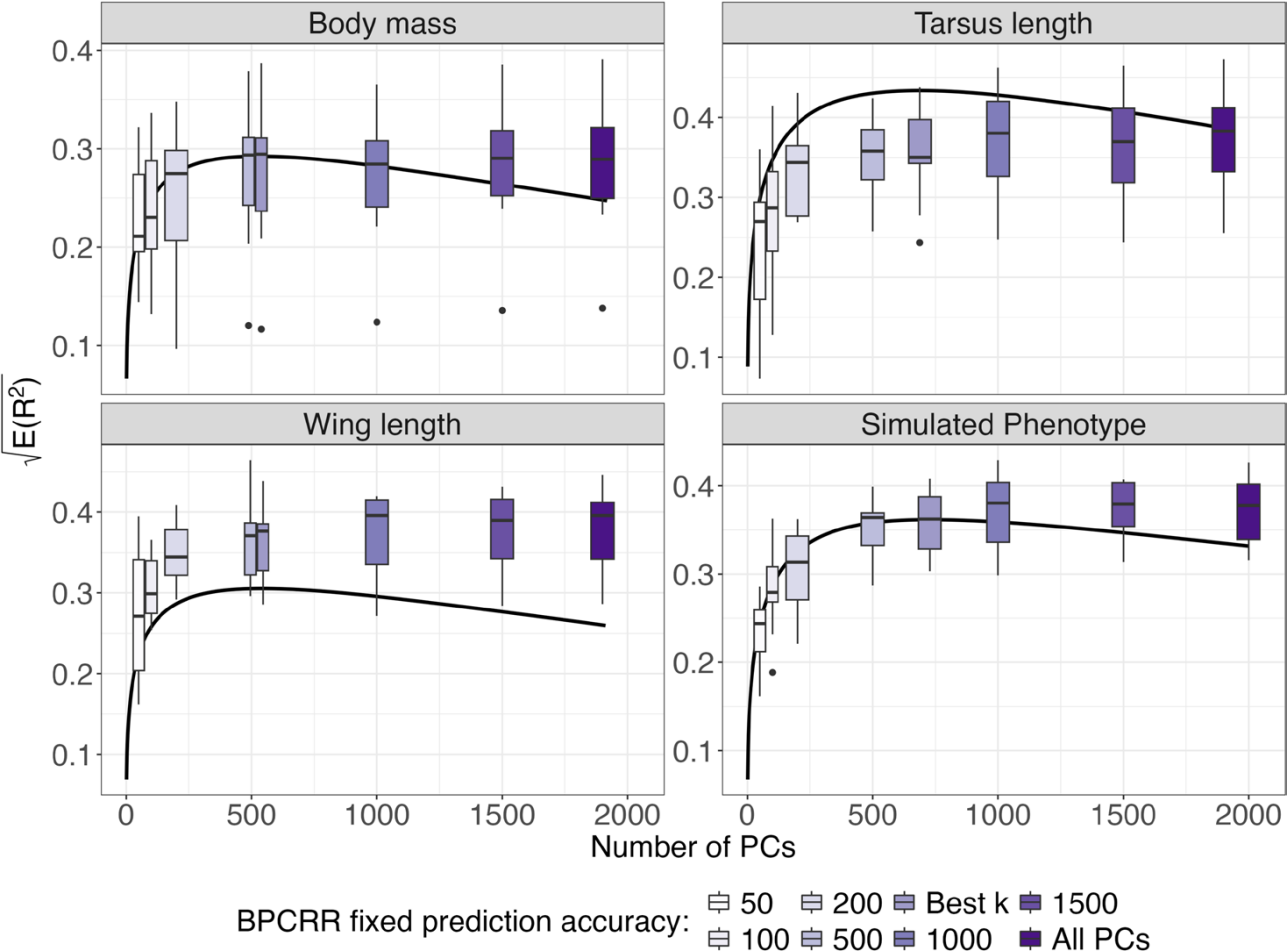
Prediction accuracy $\sqrt{E\left(R^{2}\right)}$ derived from Formulas (6) and (7), in dependence of the number of PCs (*k*) and for *h* ^2^ and *N* values that correspond to the respective real and simulated traits (black line). The actual observed prediction accuracies from 10 cross‐validation runs fitted with fixed ridge priors on the PC effects with 8 different *k*s ranging from 50 to the total number of PCs (including the “best *k*”) are added as boxplots for comparison. The observed accuracies were obtained by using the shrinkage prior discussed in the main text.

**FIGURE 3:**
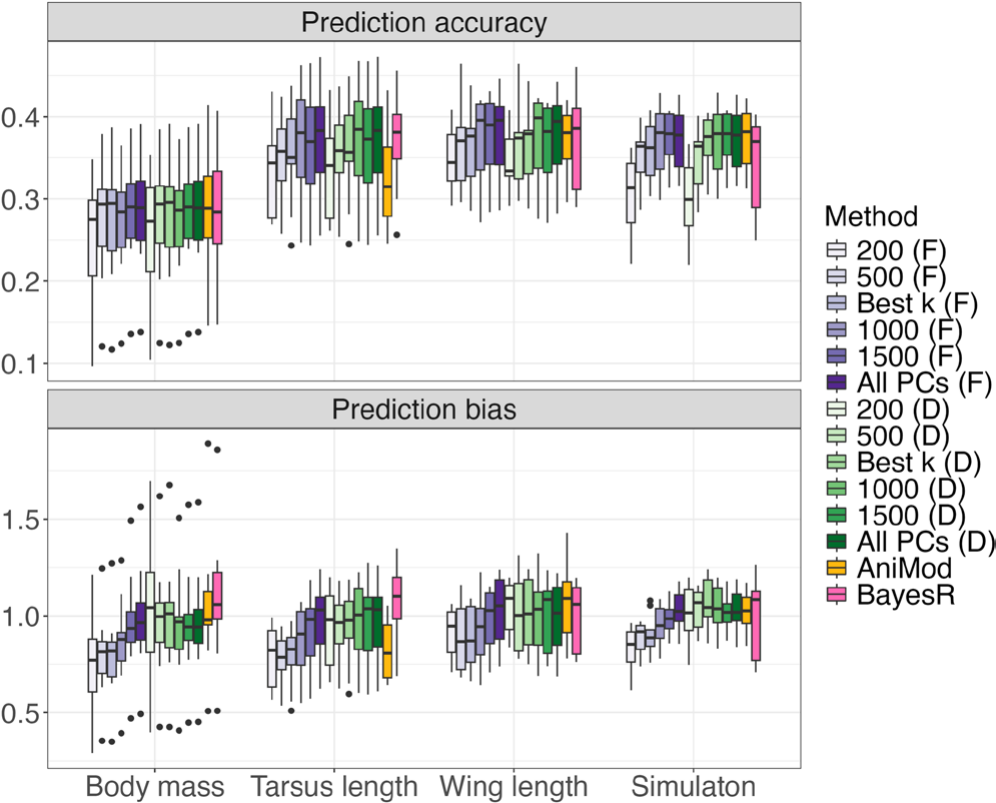
Assessment of prediction accuracy (top) and prediction bias (bottom) for BPCRR for a selection of the numbers of PCs, with *fixed* (F, in purple shades) and *default* priors (D, in green shades), as well as comparisons to the predictions from the genomic animal model (orange) and from BayesR (pink). For better visibility, the *y*‐axes were cut at 0.5 and 2.0, respectively, and therefore one case with high prediction accuracy for the genomic animal model for wing length and two cases with high prediction bias for the simulation case for BayesR are lacking.

The optimal number of PCs (k) was derived using Equation (6), along with the $h_{k}^{2}$ approximation in Equation (7). For the sparrow traits (body mass, wing length, tarsus length with $h^{2}=0.28,\;0.29,\;0.47$) and the simulated phenotype ($h^{2}=0.33$), the corresponding optimal k values were 527, 541, 688, and 728, respectively. Prediction accuracy remained high even for k exceeding the optimal value, as variance‐proportional scaling of the PCs mitigates the bias‐variance trade‐off (Figures 2 and 3). This robustness is helpful when $h^{2}$ is uncertain, or approximations in (6) and (7) are imperfect. The results were consistent using INLA's default prior (Figure S6, Appendix F). In contrast, using standard ridge scaling with a variance of 1 for all PCs led to reduced accuracy when over‐fitting the model, underlining the importance of variance‐proportional scaling.

#### 3.2.2 Assessing the Quality of Genomic Prediction

The prediction accuracy for the breeding values obtained by BPCRR was consistently high and typically equal to or slightly better than that of the GBLUPs from the genomic animal model and BayesR, regardless of whether default or fixed priors were used (Figure 3, top). In line with Section 3.2.1, BPCRR showed robust performance across a range of PC numbers: values for k between 500 and 1000 provided a stable compromise between computational efficiency (Figure S7 in Appendix G) and accuracy (Figure 3). Notably, the prediction accuracy for tarsus length was considerably lower when using the genomic animal model compared to BPCRR and BayesR, suggesting a possible violation of the underlying assumptions for the genomic animal model, such as the expectation of homogeneous and small marker effects, for this trait. As anticipated, scaling the PCs by their explained variance, rather than using standard ridge scaling, proved critical: doing otherwise led to a notable drop in prediction accuracy when k exceeded its optimal value (Figure S6 in Appendix F), providing a strong empirical confirmation of our theoretical reasoning.

In terms of bias, the slope coefficients $\beta_{1}$ from regressing mean observed phenotypes ${\bar{y}}_{i}$ against ${\hat{g}}_{i}$ showed no major deviations from 1.0 when default priors were used in BPCRR (Figure 3, bottom). When using the fixed prior from Equation (4), however, breeding values were somewhat underestimated unless nearly all PCs were included, likely due to prior‐induced shrinkage reducing variance at the expense of increased bias. Meanwhile, both the GBLUPs and BayesR showed low bias for body mass and wing length, as well as the simulated trait, but displayed downward and upward bias, respectively, for tarsus length. INLA's default priors delivered both high accuracy and low bias even with few PCs, with fixed priors mainly offering a computational advantage (Figure S7, Appendix G).

### 3.3 Analysis of Micro‐Evolution in the House Sparrow Case Study

**FIGURE 4:**
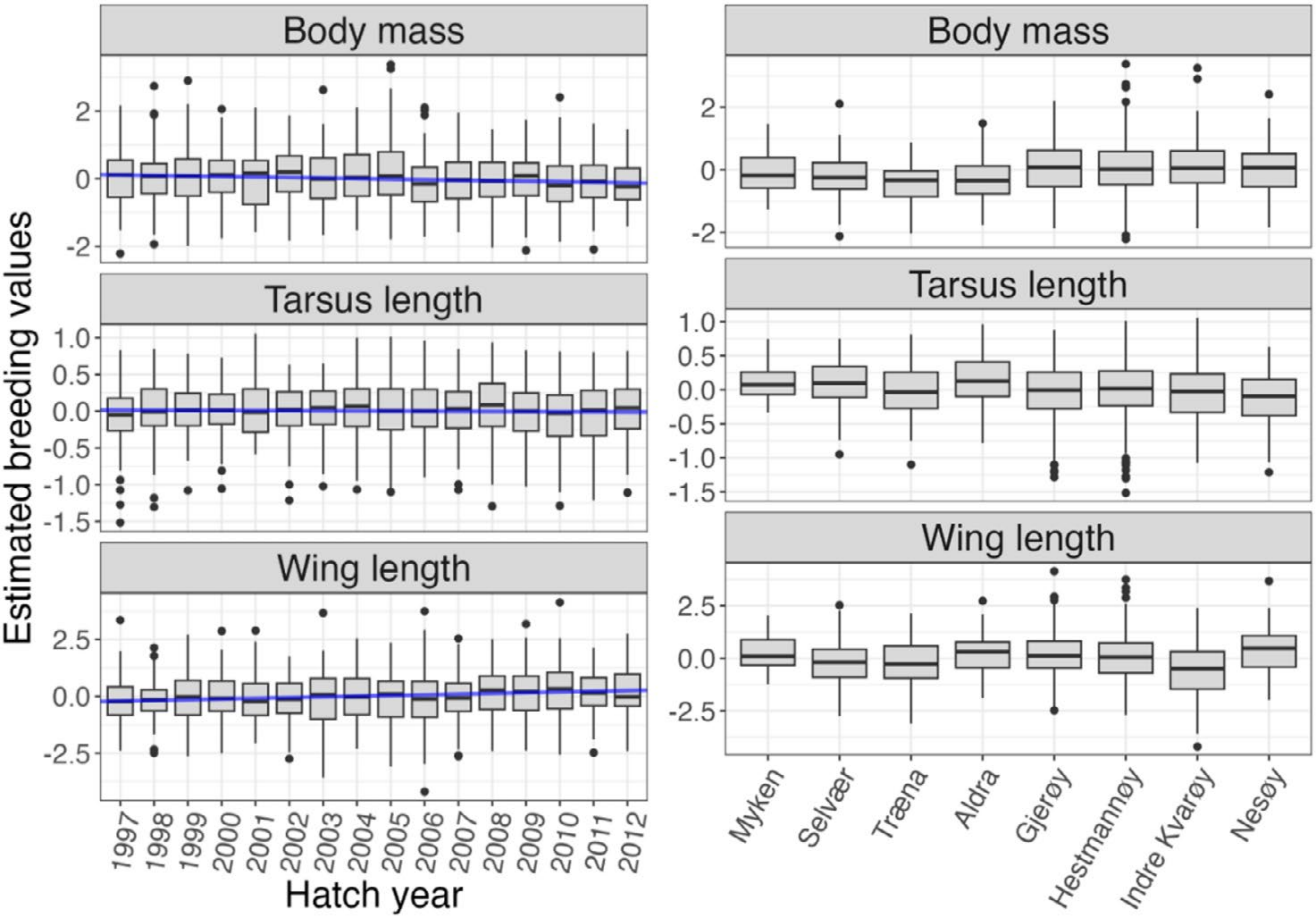
Summaries of the posterior mean breeding values derived from the BPCRR method for the three traits from the house sparrow meta‐population across birth cohorts (left) and hatch islands (right). The blue lines in the left plots represent regressions of the posterior mean breeding values on year.

The micro‐evolutionary temporal and spatial patterns using breeding values estimated from BPCRR in the house sparrow case study were biologically meaningful and consistent with existing knowledge of the system. The results revealed strong evidence that the mean breeding value for body mass decreased over generations, with an expected reduction of $\hat{\beta }=0.016$ g/year $\left(\mathrm{SE}=0.004\right)$, whereas wing length increased by $\hat{\beta }=0.031$ mm/year $\left(\mathrm{SE}=0.006\right)$. On the other hand, there was no evidence for micro‐evolutionary temporal change in tarsus length ($\hat{\beta }=-0.002$ mm/year, SE=0.002; Figure 4, left). These results are in good agreement with the full posterior distribution of slope estimates when repeatedly sampling from the posterior and regressing against year (Figure S9 in Appendix H). Interestingly, the trends in body mass and wing length were, to different degrees, also reflected in the phenotypes themselves, whereas there was evidence for a substantial phenotypic decrease in tarsus length over time, which is not reflected by the neutral micro‐evolutionary trajectory (see Table S1 and Figure S10).

On the spatial scale, we observe that some islands deviated substantially in their distribution of breeding values (Figure 4, right) compared to the general pattern for the other islands. As an example, birds on Træna seemed to be lighter and have shorter wings than most other island populations, which is in agreement with earlier observations of spatially divergent genetic and environmental patterns and possible local adaption in this system (Holand et al. 2011; Muff et al. 2019; Araya‐Ajoy et al. 2019; Aase et al. 2022).

### 3.4 Efficiency of Computations

Computation times were compared for procedures run on the same high‐performance cluster (Själander et al. 2019). Even though, in contrast to hibayes, R‐INLA is running in parallel by default, we always assigned exactly one core to fit each individual model used in this comparison for conservative benchmarking. We see a clear pattern of increasing computation times with a growing number of PCs (k) for the BPCRR method (Figure S7 in Appendix G), but the method was still faster than fitting the genomic animal model for values of k that are obtained from maximizing Equation (6) for the three traits and the simulation. For k=1000, computation times for BPCRR and the genomic animal model were comparable for the three traits, and considerably lower for BPCRR for the simulation. The last observation is in line with the fact that the computational benefits are most pronounced for the simulation, namely because the latter had larger N than the three traits from the house sparrow case study, and thus the computational benefit of the BPCRR becomes more apparent. As expected, the BPCRR method was most efficient when the shrinkage prior (4) was used, and slower for the INLA default. The BayesR implementation via the hibayes R package used here, which was chosen in order to handle the full model including all the fixed and random effects like the other two methods, was clearly the computationally least efficient. Model complexity and the choice of trait strongly affected the actual number of MCMC iterations needed until convergence (Figure S8). Further details are given in Appendix G.

## 4 Discussion

We have introduced a quantitative genetic approach for approximate estimation and prediction of key evolutionary parameters in wild systems using a framework that we call Bayesian principle component ridge regression (BPCRR). The main motivation for this development was the lack of a readily available method that can efficiently handle growing genomic data sets to perform quantitative genetics analyzes for wild study systems. Existing methods either require a two‐step approach to account for phenotypic variation in heterogeneous environments, where inbreeding, age structure, and environmental covariates may all influence the trait of interest, or can become computationally inefficient as model complexity and data size increase. BPCRR fills this gap, as it efficiently handles complex models that include an arbitrary number of fixed and random effects alongside additive genetic components derived from genomic data. Our results indicate that BPCRR is useful for both the estimation of additive genetic variance and the prediction of genomic values, although its major strength in terms of computational efficiency gains clearly lays in the latter. In fact, high prediction accuracy is retrieved even when only 25%–50% of all PCs are included.

We also assessed the biological relevance of the estimated breeding values obtained by BPCCR using the house sparrow system. When tracking micro‐evolutionary temporal and spatial patterns, we found that our results agree with earlier findings of spatially divergent genetic and environmental patterns (Holand et al. 2011; Muff et al. 2019; Araya‐Ajoy et al. 2019; Aase et al. 2022), and indicate that selection and local adaptation may be on‐going for these traits. Across the 16 birth cohorts, there were negative phenotypic trends in body mass and tarsus length, whereas wing length slightly increased phenotypically (Appendix H). Our analyzes based on BPCRR suggested that the phenotypic trends in body mass and wing length have a genetic basis, but that the decrease in tarsus length was due to changes in the environment. Given that population sizes on most islands increased quite dramatically over the study period (Niskanen et al. 2020), future studies should examine if density effects can explain the phenotypic and evolutionary changes we found in the house sparrow system. Even though stochasticity and genetic drift also act in the system (Araya‐Ajoy et al. 2025), meaning that the observed spatio‐temporal patterns in breeding values could be the results of neutral demographic and genetic processes, we have illustrated that the BPCRR method is able to accurately and efficiently estimate breeding values and $V_{A}$ for morphological traits like those investigated here.

As one of the benchmarks, we compared BPCRR to BayesR, implemented via the hibayes R package (Yin et al. 2022), which is able to handle the full model (2), in order to have a one‐to‐one comparison to the BPCRR and genomic animal model methods. However, the respective implementation using MCMC quickly becomes computationally inefficient (Figure S2). BayesR is therefore often employed as a two‐step procedure, where residuals from a pre‐fitted linear mixed model are subsequently analyzed (Ashraf et al. 2022). An under‐recognized problem is that such a two‐step approach can substantially underestimate $V_{A}$, particularly when residual variance is high (Figure S1). Preliminary results indicate that combining individual‐specific effects with the averaged residual over repeated measures as a new response could alleviate this bias, although further investigations are needed (Appendix A). Another, principal advantage of integrated modelling frameworks such as BPCRR lies in their capacity to propagate uncertainty consistently across all model components, an aspect often compromised in two‐step approaches. Moreover, BPCRR can easily accommodate interaction terms, in particular genotype‐by‐environment (G×E) effects. Accurate modelling of such interactions is increasingly recognized as essential for understanding the genetic basis of trait variation in heterogeneous environments, which are pervasive across wild, domesticated, and human systems (Wang et al. 2019; Christensen et al. 2021; Di Leo et al. 2022).

As sample sizes and marker densities continue to expand in studies of wild populations, computational efficiency becomes an increasingly important criterion. In Bayesian setups, the choice of sensible priors often considerably impacts the computational cost. In fact, we found that the use of informative shrinkage priors substantially accelerates computation in BPCRR, particularly as the number of individuals (N) increases. At the same time, the naïve prior retains the advantage of yielding unbiased genomic predictions even when relatively few principal components are included (Figure 3). Importantly, the number of principal components required for accurate genomic prediction does not scale linearly with N in homogeneous populations, which is underpinned by Formula (6), and illustrated by our empirical analyzes and simulation studies. As a consequence, the relative computational advantage of BPCRR over genomic animal models becomes increasingly pronounced as both N and the number of markers (m) grow (Figure S7 in Appendix G). While Formula (6) provides a practical, approximate lower bound for determining the number of PCs required, it rests on simplifying assumptions, such as random mating and the absence of environmental or population structure (Wray et al. 2013, 2019; Aase et al. 2025). Although the formula proved robust within our dataset, population structure is common (Jombart et al. 2009; Luu et al. 2017), and some of those assumptions are indeed clearly violated in our system (see for example Appendix C). Further refinement of the underlying theory to incorporate structured populations and environmental covariates would therefore broaden its applicability (Dekkers et al. 2021).

Previous studies indicate that the performance of genomic prediction methods depends on the genetic architecture of the analyzed trait (e.g., Moser et al. 2015; Meher et al. 2022). Here, BPCRR proved highly effective for polygenic traits, where the variance captured by principal components approximately scales linearly with the proportion of variance explained by the PCs that are included in the model (Figure S3, Appendix B). Simulations further demonstrated that BPCRR maintains predictive performance comparable to that of BayesR even for traits with oligogenic genetic architectures, provided that causal variants are relatively uniformly distributed across the genome (Figure S5, Appendix D). The same is true for the genomic animal model, which is based on the infinitesimal model assumption, implying that the marker effect sizes u all stem from the same distribution (Meuwissen et al. 2016). Importantly, however, BPCRR does not impose such a homogeneity assumption, and marker‐specific effects can be recovered post hoc by back‐transforming PC effects (Ødegård et al. 2018), offering flexibility for downstream analyzes. It is thus not surprising that BayesR and BPCRR showed similar prediction accuracy for tarsus length (Figure 3), which is a skeletal trait that potentially is less polygenic than body mass and wing length (Hansson et al. 2018; Yengo et al. 2022), while the GBLUPs obtained from the genomic animal model were less precise in this case. A similar observation was made for the two skeletal traits “foreleg length” and “horn length” in a study of wild Soay sheep (Ashraf et al. 2022).

The field of genomic prediction has so far mainly been driven forward by animal and plant breeders, as well as by researchers in human genomics (Meuwissen et al. 2001; Wray et al. 2007, 2019). However, the growing volume and complexity of genomic data sets in wild systems demands statistical approaches that are computationally efficient and at the same time flexible and robust, so that they can help us answer important questions related to evolutionary processes in the wild. Thanks to a combination of efficiency and the possibility to jointly model any genetic and environmental effects without the need for a two‐step procedure, extensions such as the modelling of dominance variance and G×E interactions, are straightforward within the BPCRR framework, further enhancing its applicability to the study of eco‐evolutionary processes. We thus see BPCRR as a complementing alternative and a promising approach that fills an opening gap to handle current and future challenges of wild study systems.

## Funding

This study was partially funded by the European Union (ERC, GPWILD, project number 101169862). Views and opinions expressed are however those of the author(s) only and do not necessarily reflect those of the European Union or the European Research Council. Neither the European Union nor the granting authority can be held responsible for them. This study was also funded by the Norwegian Research Council (project numbers 191847, 221956, 274930 and 302619). The Research Council of Norway also partly supported this work through its Centres of Excellence funding scheme (project number 223257). Genotyping on the custom house sparrow Axiom 200 K SNP array was carried out at CIGENE, Norwegian University of Life Sciences, Norway. Computations were performed on resources provided by the NTNU IDUN/EPIC computing cluster (Själander et al. 2019).

## Conflicts of Interest

The authors declare no conflicts of interest.

## Supporting information


**Appendix A:** Two‐step procedure in BayesR.
**Appendix B:** Proportionality of explained variances.
**Appendix C:** Effects of SNP and PC scaling on genomic clustering PCA.
**Appendix D:** Simulation for different genetic architectures.
**Appendix E:** Computational details and software.
**Appendix F:** Genomic prediction accuracy and its dependence on priors and scaling.
**Appendix G:** Efficiency of computations.
**Appendix H:** Supplementary figures for the analysis of micro‐evolutionary change in the house sparrows.

## Data Availability

The data and code that support the findings of this study are openly available in Dryad at http://datadryad.org/share/d‐zldiQko5xPQQTDTujAieYK1b2smXi6LTsyuvNYV9c.
