# Supplementary material for: A Flexible Method for Genomics‐Based Quantitative Genetics in Wild Study Systems—A Case Study on a House Sparrow Meta‐Population
Source: Evol Appl. 2026 Apr 27;19(4):e70242. doi: 10.1111/eva.70242 (PMC13112192; doi:10.1111/eva.70242)
Supplement: Supplementary file 1 — Appendix A: Two‐step procedure in BayesR. Appendix B: Proportionality of explained variances. Appendix C: Effects of SNP and PC scaling on genomic clustering PCA. Appendix D: Simulation for different genetic architectures. Appendix E: Computational details and software. Appendix F: Genomic prediction accuracy and its dependence on priors and scaling. Appendix G: Efficiency of computations. Appendix H: Supplementary figures for the analysis of micro‐evolutionary change in the house sparrows. [file EVA-19-e70242-s001.pdf]

A flexible method for genomics-based quantitative genetics in wild study systems – A case study on a house sparrow meta-population

January 21, 2026

Appendix A: Two-step procedure in BayesR

For comparison, we ran a 10-fold cross-validation for each trait, and estimated posterior distributions for V_A from the full data set using the BayesR v2 software package based on Moser et al. (2015). We used Dirichlet priors with 1, 1, 1, 5 and default values otherwise. All MCMC chains used to generate Figure S1 were run for 5000 iterations, a burn-in of 1000 and a saving frequency of 10. In all cases, computation time was between 1.5 and 3 hours on personal laptop with an Intel Core i7-1260P CPU. Note that these are relatively short chains compared to those generated by `hibayes` in the main text. Visual convergence checks indicate that the chains are stationary, but should be run 5-10 times longer if the aim was to derive reliable inference.

Here, the aim was mainly to illustrate the consequence of using a two-step procedure with BayesR (Figure S1). In a two-step approach, a linear mixed model (LMM) is first fitted with all the fixed and random effects except the genetic value. The estimated random individual-specific (ID) effect from this pre-fitted LMM is then used as the new response. However, while such a procedure leads to good prediction accuracy, given as the correlation between predicted breeding value and mean phenotype (Figure S1, top), it underestimates the actual variance in the genetic values, that is, V_A (Figure S1, bottom a) - c)). The problem is particularly pronounced for body mass and wing length, where using the sum of the ID effect plus the mean of the residuals over all repeats within an individual (ID+res) recovers a correct V_A estimate that is in line with the genomic animal model, BPCRR and BayesR (see main text, Figure 1). Both body mass and wing length are plastic traits, thus the residuals absorb part of the within-individual variability of the genetic value. Tarsus length, on the other hand, is static skeletal trait and seems not (or much less) affected by the observed effect.

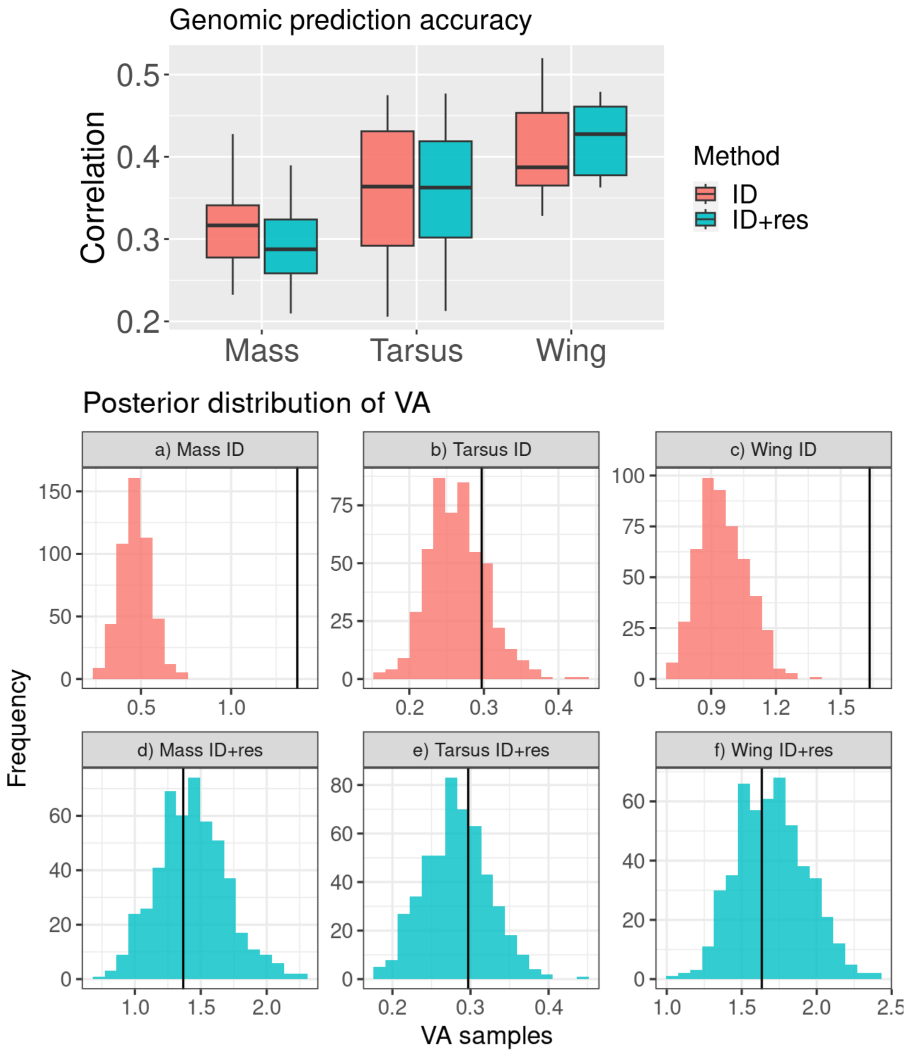

Figure S1: Comparison of the results for the two-step implementation of BayesR for the cases where the individual-specific ID effect is used as the new response in the second step (denoted as ID in the figure), and the case where we take the sum of the ID effect plus the mean of the residuals over all repeats within an individual (denoted as ID+res). The results from a 10-fold cross-validation illustrate that prediction accuracy tends to be higher when using ID as the new response (top), in particular for the two plastic traits mass and wing. The correlation was calculated between the predicted breeding value and the mean observed phenotype per individual. However, V_A then tends to be underestimated for those traits (bottom), while using ID+res, recovers correct V_A. Vertical black lines indicate the estimated V_A value from the genomic animal model.

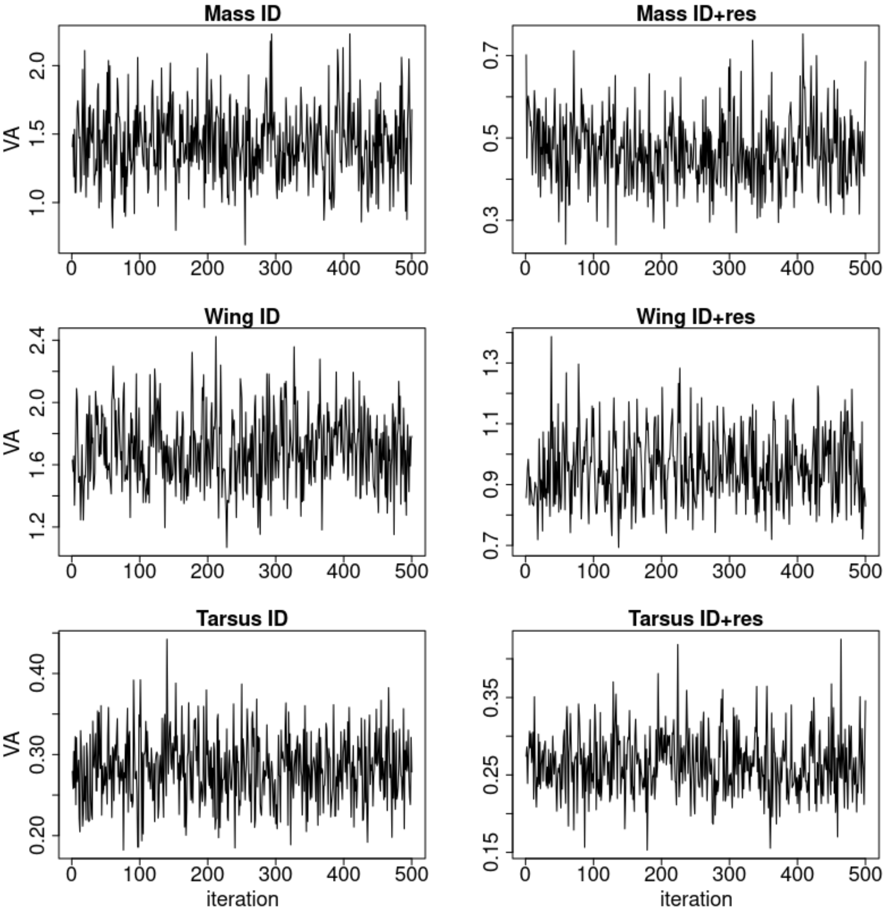

Figure S2: Trace plots for the six models analyzed with a two-step procedure, using the BayesR v2 software. Given that the thinning was 10, only every 10th MCMC iteration is shown.

Appendix B: Proportionality of explained variances

If the infinitesimal model assumption holds, that is, for highly polygenic traits, we expect the variance explained by a PC to be linearly related to the variance it explains in the breeding value of the trait of interest. Another direct implication of the infinitesimal model assumption is that there is a linear correspondence between the proportion of variance explained by the first k PCs and the proportion of additive genetic variance explained in the trait of interest, which in turn corresponds to the relation between the estimated and true heritabilities $\frac{h_k^2}{h^2}$ obtained from using the k first PCs. The approach for choosing the optimum number of PCs in fact relies on this assumption. Figure S3 indicates that the respective assumption is approximately fulfilled.

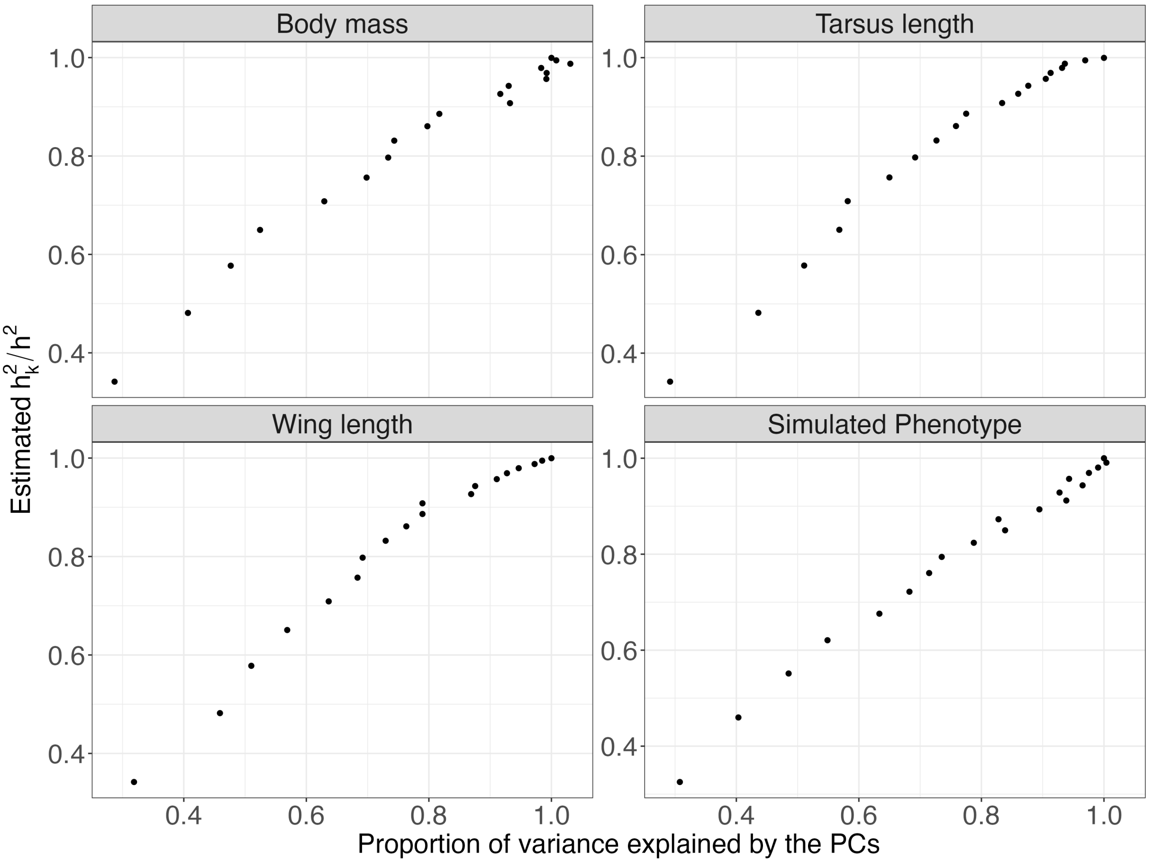

Figure S3: Relation between the proportion of estimated heritabilty $\frac{h_k^2}{h^2}$ against the proportion of variance explained by the respective set of PCs. The results for the three traits (body mass, wing length and tarsus length) and the simulated phenotype indicate that the relationship is approximately linear, reflecting that the variance explained by a PC can be assumed approximately linearly related to the variance it explains in the breeding value of the trait of interest.

Appendix C: Effects of SNP and PC scaling on genomic clustering PCA

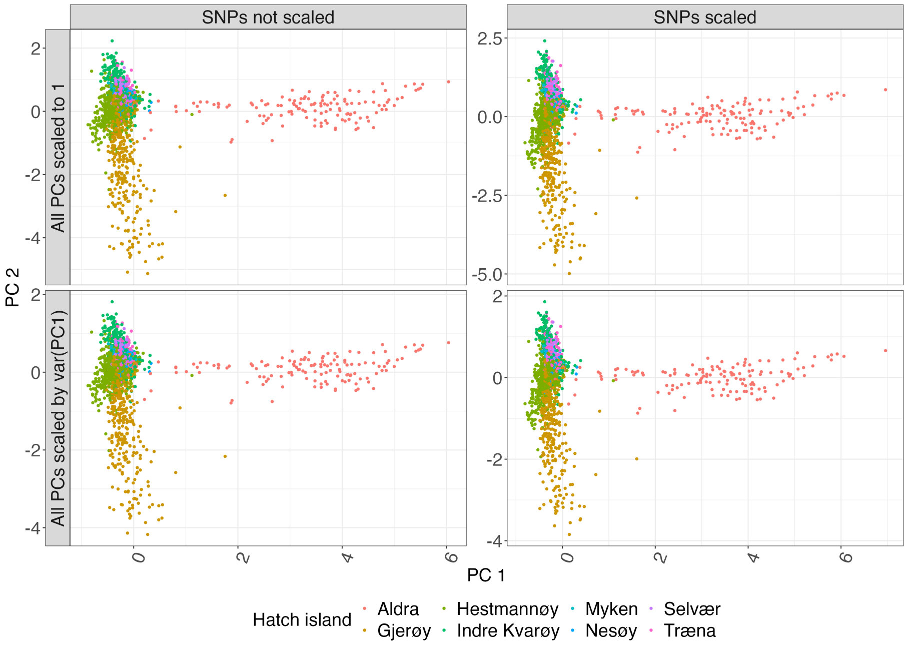

Figure S4: Genomic clustering based on the first two principal components (PC1 and PC2) under alternative SNP and PC scaling schemes. Columns show results obtained using unscaled SNPs (left) and SNPs scaled to unit variance (right). Rows show PCs scaled to unit variance (top) and PCs scaled relative to the variance of the first PC (bottom). Points represent individuals and are coloured according to hatch island. Differences among scaling schemes primarily affect the scale of the PC axes, while the overall spatial structure of the meta-population is preserved

Appendix D: Simulation for different genetic architectures

We designed a small and simple experiment to investigate how different genetic architectures affect genomic prediction accuracy. To this end, we used five different distributions (denoted as D1 to D5), where each distribution was a mixture of only two components: A component with zero effect, and a normally distributed component $u_j \sim \mathsf{N}(\mathbf{0}, \sigma^2)$, that is,

$$u_j \sim \pi_1 \cdot 0 + \pi_2 \mathsf{N}(0, \sigma^2) \ .$$

The weight π_2 of the normally distributed component was thereby chosen as 100%, 50%, 25%, 5%, and 0.5% for D1, D2, D3, D4, and D5, respectively. As in the main text, the breeding values were obtained as linear combinations of all the SNP values, and scaled such that $\sigma_G^2 = 0.33$. Again, the normally distributed residuals added up to a total phenotypic variance of 1. For all five distributions of marker effects, we evaluated the prediction accuracy of BPCRR for a variety of number of PCs, and compared the results to the genomic animal model and BayesR (Figure S5). The main observation is that, despite different assumptions on the marker effects, the three methods always yield similar prediction accuracy within a genetic architecture. Keep in mind that the results in Figure S5 stem from only one trial for each effect size distribution, thus the actual level of accuracy varies between the distributions.

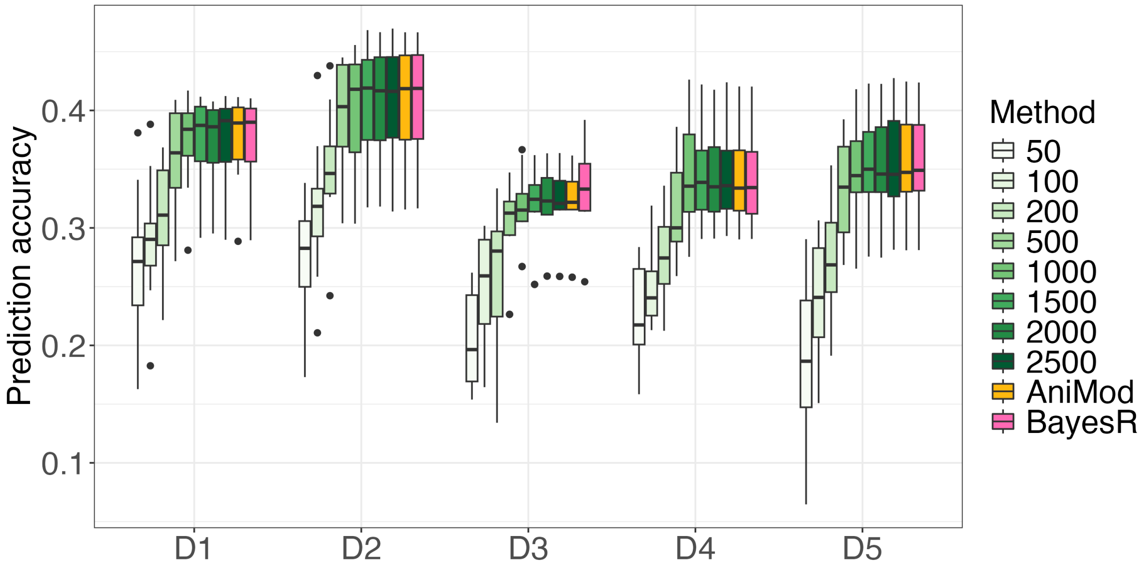

Figure S5: Prediction accuracy for distributions D1 (with $\pi_2 = 1$) to D5 (with $\pi_2 = 0.005$) for selected numbers of k between 50 and 2500 for BPCRR using the *default* prior, as well as comparisons to the genomic animal model (AniMod) and BayesR. In each case, a 10-fold cross-validation was carried out and the results are aggregated in the boxplots.

Appendix E: Computational details and software

All analyses for the genomic animal model and BPCRR were performed with version 20.3.17 of the the `R-INLA` R package (Martins et al., 2013; Rue et al., 2017) in the statistical software R (R Core Team, 2024) version 4.3.1. The GRM for the genomic animal model was derived using the van Raden-method (VanRaden et al., 2009) implemented in the AGHmatrix package version 2.0.4 (Rampazo Amadeu et al., 2016). The SVDs needed in BPCRR were derived with the function svds() from the Rspectra R package (Qiu and Mei, 2019), as demonstrated the coded example for a simulated case that we provide on the Dryad repository for this article. In applications where not all individuals had measured phenotypes, the SVD was done for the respective sub-matrix including SNP data only from the phenotyped individuals that were included in the model, since the inclusion of additional individuals in the SVD calculation would otherwise introduce unwanted and unnecessary noise into the PCs. For the BayesR analyses we used the package `hibayes` (Yin et al., 2022) version 3.0.0 in the R-version 4.2.1. The length of the burn-in and total number of iterations for the MCMC chains in `hibayes` were selected for each case according to visual inspection of the convergence plots, in order to ensure a good trade-off between accuracy and computational time. The thinning interval was set to 10.

Unless stated otherwise, all analyses were performed on a local high-performance computing cluster (Själander et al., 2019).

Appendix F: Genomic prediction accuracy and its dependence on priors and scaling

Figure 2 in the main text shows genomic prediction accuracy depending on the number of PCs for the case where the informative point priors from formula (4) were used. Importantly, the results look almost identical for the case where no prior knowledge on σ_G^2 was assumed, that is, when default priors $\sigma_{u^\star}^2 \sim \Gamma(1, 5 \cdot 10^{-5})$ for the variance of the PC effects were used (Figure S6, green boxplots).

In addition, we have also compared different ways to scale the variances of the PCs. As described in the main text (Sections 2.2.2 to 2.2.3), we propose to scale the PC variances such that they remain proportional to their eigenvalues (Macciotta et al., 2010). A major benefit of not standardizing the variances to unity – which corresponds to the common ridge regression standardization for arbitrary predictors – is that even including large numbers of PCs then typically does not lead to significantly lower prediction accuracy than for the "best" k found by equation (6) in the main text, namely because PC-effects for PCs with less variance are automatically shrunken more, and over-fitting is omitted (Figure S6, green boxplots). In contrast, here we illustrate how standardizing the variances of all PCs to unity leads to a prediction accuracy decreases after the optimal balance is reached, as expected (Figure S6, purple boxplots).

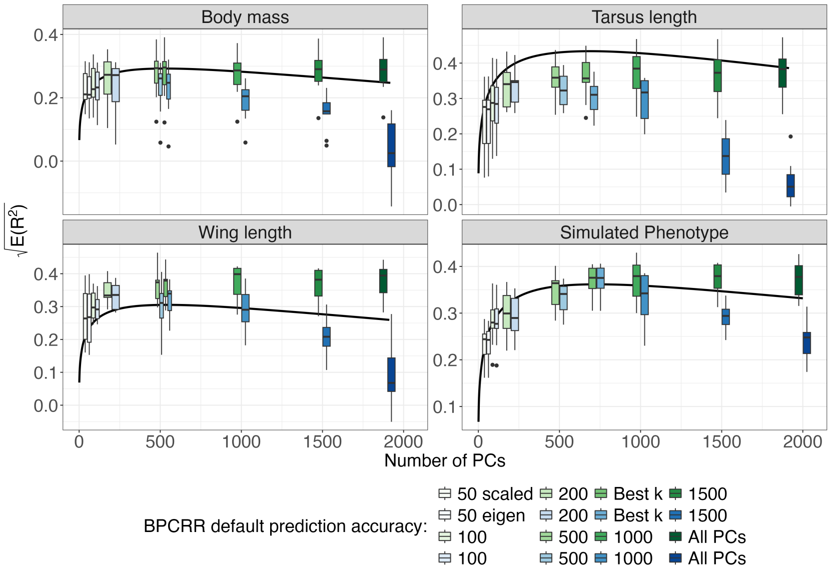

Figure S6: Comparison of the expected prediction accuracy $\sqrt{E(R^2)}$ using formulas (6) and (7), in dependence of the number of PCs (k) and for h^2 and N values that correspond to the respective cases (black line), and the actually observed prediction accuracies from a 10-fold cross-validation, represented as boxplots (in green). The figure are enriched with results where all PCs are scaled to 1 (purple). The observed accuracies were obtained by using default priors in INLA.

Appendix G: Efficiency of computations

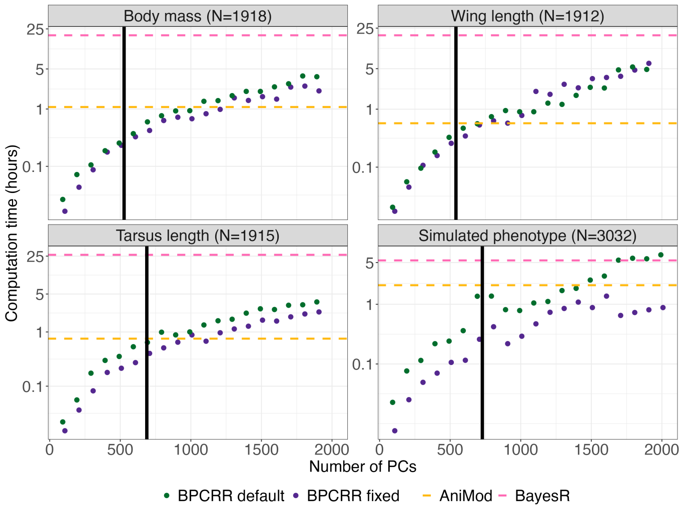

Figure S7: Computation times for BPCRR (fixed and INLA default priors, in purple and green, respectively), BayesR (in pink) and the genomic animal model (AniMod, in orange). The black vertical lines indicate the number of PCs obtained from maximizing equation (6). Note that the y-axis is on log-scale, thus a linear increase in the graph corresponds to an exponential increase in absolute values.

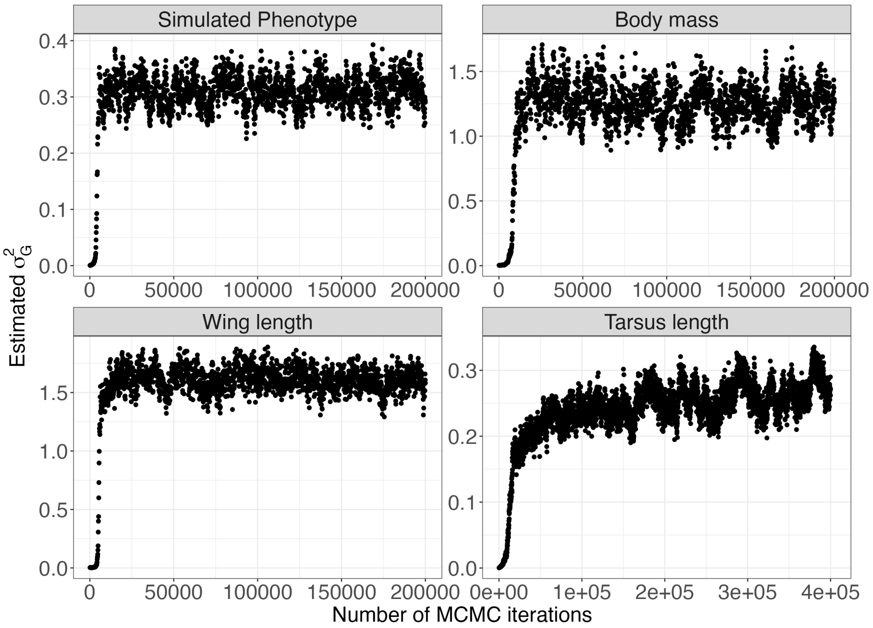

Figure S8: Convergence plots for simulated data and the traits for the longest chains that were run on the full dataset, with one data point for every hundred MCMC iterations. Note that tarsus length shows very slow convergence, and that even 400'000 iterations are a lower limit for this trait.

Model complexity and the choice of trait strongly affected the actual number of

MCMC iterations needed until convergence. While convergence for the MCMC chains occurred relatively quickly for the simulated phenotype, wing length and body mass, more samples were needed in the case of tarsus length, as indicated by the convergence plots that were run on the full datasets for long enough until we observed convergence (Figure S8). Based on the convergence plots, we chose a burn-in of 25 000 and a sample size of 50 000 for body mass and wing length, a burn-in of 100 000 and 50 000 samples was used for tarsus length. Finally a burn-in of 10 000 and a sample size of 20 000 for the simulation study, which, unsurprisingly, was the least problematic in terms of convergence. Importantly, we would like to stress that the computational burden is similar for the commonly used implementation of the BayesR procedure (Moser et al., 2015; Ashraf et al., 2022; Hunter et al., 2022), which is based on the two-step approach mentioned in Section 2.1.3 (see Appendix A).

Appendix H: Supplementary figures for the analysis of micro-evolutionary change in the house sparrows

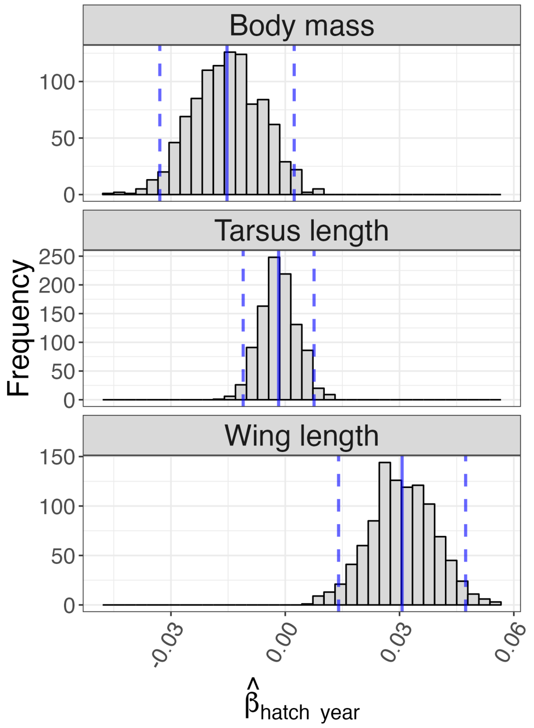

Figure S9: Distribution of slope estimates when repeatedly sampling from the posterior distribution of breeding values for each indidivual and regressing against hatch year. The blue solid and dashed lines mark slope estimates and 95% confidence intervals, respectively, for the results given in the main text, that is, when posterior mean breeding values for each individual are directly regressed on time, instead of repeatedly sampled breeding values. These results illustrate the consistency between the two approaches.

	$\hat{\beta}$ (SE; p-value)<br>y=Estimated breeding value	$\hat{\beta}$ (SE, p-value)<br>y=Measured henotype
**Body mass**	-0.016 (0.004; $p < 0.001$)	-0.030 (0.011; $p = 0.008$)
**Tarsus length**	-0.002 (0.002; $p = 0.45$)	-0.024 (0.004; $p < 0.001$)
**Wing length**	0.031 (0.006; $p < 0.001$)	0.015 (0.012; $p = 0.19$)

Table S1: Estimated regression slopes ($\hat{\beta}$), standard errors (SE) and p-values for the linear regression against hatch year, both for the case where the breeding value was used as the response (left), and when the measured phenotype was the response.

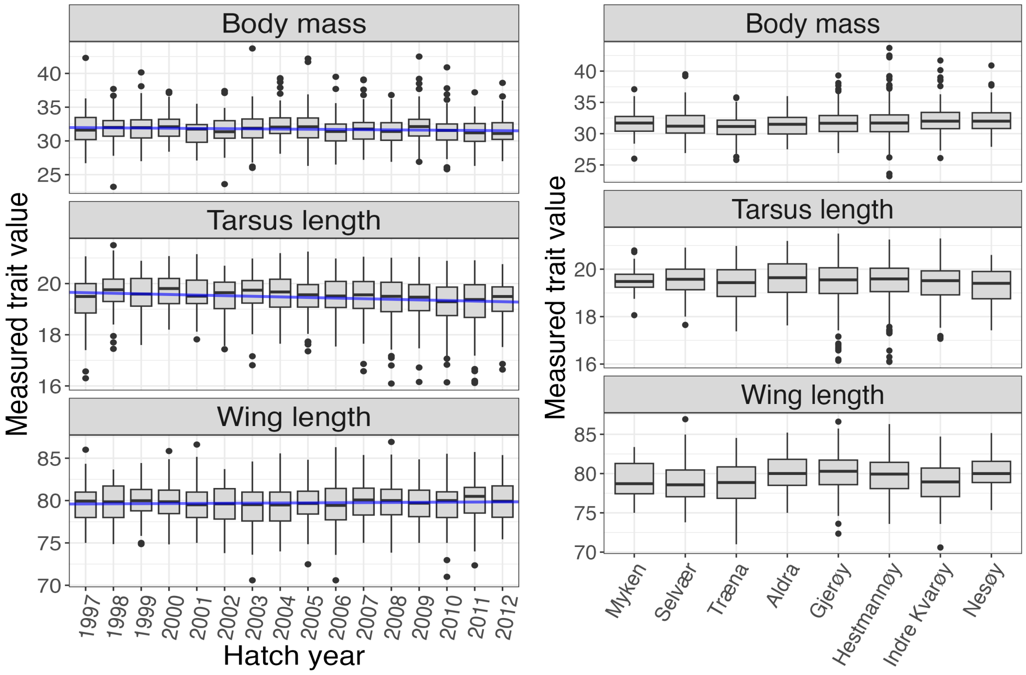

Figure S10: Summaries of phenotypic measurements for the three traits from the house sparrow case study across birth cohorts (left) and hatch islands (right).

References

Ashraf, B., D. C. Hunter, C. Bérénos, P. A. Ellis, S. E. Johnston, J. G. Pilkington, J. M. Pemberton, and J. Slate (2022). Genomic prediction in the wild: a case study in Soay sheep. *Molecular Ecology 31*, 6541–6555.

Hunter, D. C., B. Ashraf, C. Bérénos, P. A. Ellis, S. E. Johnston, A. J. Wilson, J. G. Pilkington, J. M. Pemberton, and S. J. (2022). Using genomic prediction to detect microevolutionary change of a quantitative trait. *Proceedings of the Royal Society B 289*, 20220330.

Macciotta, N. P. P., G. Gaspa, R. Steri, E. L. Nicolazzi, C. Dimauro, C. Pieramati, and A. Cappio-Borlino (2010). Using eigenvalues as variance priors in the prediction of genomic breeding values by principal component analysis. *Journal of Dairy Science 93*, 2765–2774.

Martins, T. G., D. Simpson, F. Lindgren, and H. Rue (2013). Bayesian computing with INLA: New features. *Computational Statistics and Data Analysis 67*, 68–83.

Moser, G., B. J. Lee, S. H. Hayes, M. E. Goddard, N. R. Wray, and P. M. Visscher (2015). Simultaneous discovery, estimation and prediction analysis of complex traits using a Bayesian mixture model. *PLoS Genetics 11*, e1004969.

Qiu, Y. and J. Mei (2019). *RSpectra: Solvers for Large-Scale Eigenvalue and SVD Problems.* R package version 0.16-0.

R Core Team (2024). *R: A Language and Environment for Statistical Computing.* Vienna, Austria: R Foundation for Statistical Computing.

Rampazo Amadeu, R., C. Cellon, J. W. Olmestead, A. A. Franco Garcia, and M. F. Resende Jr (2016). AGHmatrix: R package to construct relationship matrices for autotetraploid and diploid species: a blueberry example. *The Plant Genome 9*, 1–10.

Rue, H., A. I. Riebler, S. H. Sørbye, J. B. Illian, D. P. Simpson, and F. K. Lindgren (2017). Bayesian computing with INLA: a review. *Annual Reviews of Statistics and Its Applications 4*, 395–421.

Själander, M., M. Jahre, G. Tufte, and N. Reissmann (2019). EPIC: An energy-efficient, high-performance GPGPU computing research infrastructure. *arXiv preprint arXiv:1912.05848*.

VanRaden, P., C. Van Tassell, G. Wiggans, T. Sonstegard, R. Schnabel, J. Taylor, and F. Schenkel (2009). Invited review: reliability of genomic predictions for North American Holstein bulls. *Journal of Dairy Science 92*, 16–24.

Yin, L., H. Zhang, and X. Liu (2022). *hibayes: Individual-Level, Summary-Level and Single-Step Bayesian Regression Model.* R package version 1.0.1.
